# A Systematic Review of Systematic Reviews and Panoramic Meta-Analysis: Staples versus Sutures for Surgical Procedures

**DOI:** 10.1371/journal.pone.0075132

**Published:** 2013-10-07

**Authors:** Karla Hemming, Thomas Pinkney, Kay Futaba, Mary Pennant, Dion G. Morton, Richard J. Lilford

**Affiliations:** 1 Public Health, Epidemiology and Biostatistics, University of Birmingham, Birmingham, United Kingdom; 2 School of Cancer Sciences, University Hospital Birmingham, NHS Foundation Trust, Queen Elizabeth Hospital, Birmingham, United Kingdom; Harvard Medical School, United States of America

## Abstract

**Objective:**

To systematically evaluate the evidence across surgical specialties as to whether staples or sutures better improve patient and provider level outcomes.

**Design:**

A systematic review of systematic reviews and panoramic meta-analysis of pooled estimates.

**Results:**

Eleven systematic reviews, including 13,661 observations, met the inclusion criteria. In orthopaedic surgery sutures were found to be preferable, and for appendicial stump sutures were protective against both surgical site infection and post surgical complications. However, staples were protective against leak in ilecolic anastomosis. For all other surgery types the evidence was inconclusive with wider confidence intervals including the possibly of preferential outcomes for surgical site infection or post surgical complication for either staples or sutures. Whilst reviews showed substantial variation in mean differences in operating time (I^2^ 94%) there was clear evidence of a reduction in average operating time across all surgery types. Few reviews reported on length of stay, but the three reviews that did (I^2^ 0%, including 950 observations) showed a non significant reduction in length of stay, but showed evidence of publication bias (P-value for Egger test 0.05).

**Conclusions:**

Evidence across surgical specialties indicates that wound closure with staples reduces the mean operating time. Despite including several thousand observations, no clear evidence of superiority emerged for either staples or sutures with respect to surgical site infection, post surgical complications, or length of stay.

## Introduction

There are more than 6,000,000 surgical procedures performed each year in England alone [1]. Many non-modifiable factors are associated with poor surgical outcomes, including age, pre-existing co-morbidities and type of surgery [2]. However, there are also potentially modifiable factors which are associated with poor outcomes such as surgical site infection, wound dehiscence and other post-surgical complications [3]. Whilst each of these outcomes are treatable for the most part, in a significant minority they lead to further difficult to treat complications, such as scaring and pain [4] and in some cases complications might not respond to treatment and consequently lead to death [2], [5]. Post-surgical complications, including infection, lead to increases in length of stay, additional treatments and care, and so are consequently costly for health care providers [6], [7]. In light of this, a surgical evidence base, as a means to reduce the impact of surgical site infection and post surgical complications, are ever increasingly recognised as being important.

Many views have been expressed on whether sutures or staples are associated with lower rates of surgical site infection and complications; whilst staples are widely believed to result in decreased operating time [8]–[15]. However these widely held beliefs are not necessarily based on an evidence-based framework. Guidelines by NICE in 2008 on surgical site infection identified 11 randomised controlled trials in 8 different surgery types, which had compared staples and sutures [16]. The guideline found no evidence of a difference between the two methods of closure in rates of surgical site infection (the only outcome considered) although it cautioned that more primary randomised controlled trials were needed. Further to this, additional primary trials have been reported and, importantly, evidence has begun to be synthesised within surgery types and so, for example, there are now published systematic reviews of staples and sutures for closure after caesarean section and after orthopaedic surgery [17], [18]. However, publication of one of these recent systematic reviews initiated a great deal of debate and consensus on the relative merits of staples and sutures has not been reached [8]–[15].

Current systematic reviews, within surgery types, provide an evidence basis for that surgery type only. However, in some surgery types no systematic review currently exists and there may be few or even no trials. In addition, even within those surgical specialties for which systematic reviews exist, the evidence is often not conclusive, due to small study sizes, small numbers of studies and poor quality studies. Whilst undoubtedly more weight must be given to evidence from within a particular surgical specialty, where no such evidence exists, then it is natural for clinicians to consider the issue across the broader spectrum of different specialties. To this end, a review of evidence across specialties (i.e. surgery types) can sometimes be warranted. This might consist of a narrative or informal review. Alternatively, it might consist of a quantitative analysis across a systematic review of systematic reviews [19].

A systematic review of systematic reviews is a means of summarising current evidence across specialities of the same or very similar intervention, to provide a synthesis of treatment effects [20], [21]. This method does not necessarily involve pooling treatment effects, but might do using the methods of panoramic meta-analysis [22]. A panoramic meta-analysis is a means of pooling estimates across systematic reviews to obtain an overall (over specialties and studies) estimate of treatment effect [22]. A panoramic meta-analysis allows for both between review variation and between study variation, as opposed to just between study variation as in a typical meta-analysis. These methods have been used to compare the use of prophylactic antibiotics in “clean” and “dirty” surgeries [20] and to compare the efficacy of adjuvant chemotherapy across different types of carcinoma [21].

Our objective was to systematically identify all systematic reviews comparing staples and sutures across all surgical specialties. Both clinical and process outcomes were evaluated, including surgical site infection, post surgical complications, operating time and length of stay. Our primary intention was to provide a synthesis of results across surgical specialities. A secondary aim was to provide a pooled estimate of effect across surgical specialties, provided the degree of heterogeneity between specialties was moderate.

## Methods

### Search Strategy for Identification of Systematic Reviews

A protocol was drafted before implementation of the review (a copy is available from the authors). Searches were conducted of Medline (via pubmed), EMBASE and the Cochrane library (which includes the DARE database of abstracts of reviews of interventions), on 15th December 2011 (Table 1) and included a combination of free text and MeSH terms. Only peer reviewed systematic reviews published after 1980 were included. Searches were limited to *meta-analysis*, *systematic reviews or review* type publications. No language restrictions were imposed. The title and abstract of each article was scanned (independently by two reviewers: KH and either TP or KF) and full copies of articles of potentially eligible reviews were obtained. Potentially eligible reviews were then screened, again independently by two reviewers, according to the review selection criteria outlined below. All resulting references were screened for identification of additional reviews.

**Table 1:** Search strategies.

Database	Search Terms
Medline	#1 sutures or suture technique[MeSH Major Topic]
	**#2 review[Publication Type] OR meta analysis[Publication Type]**
	Limits 1980 onwards
EMBASE	1980 to 2011 December 14
	#1(review or meta analysis or systematic review).pt.
	#2(stapl$ or sutur$ or handsewn or hand sewn or skin closure or wound closure).ti.
	#3(sutures or suture techniques or surgical stapling or surgical staplers).
	#4(sutures or suture techniques or surgical stapling or surgical staplers).sh.
	#4b (wound infection or wound dehiscence).kw.
	#5 1 and 2
	#6 1 and 3
	#7 1and 4
	#8 1 and 4b
	5 or 6 or 7 or 8
Cochrane Library	(stapl* or sutur* or **handsewn or “hand sewn” or “skin closure” or “wound closure” or “wound infection” or “wound dehiscence”) in Title, Abstract or Key Word**
	**Limits: Restricted to the Cochrane Reviews and Other Reviews And from 1980 onwards**

### Review Selection Criteria

Only systematic reviews were included. Case reports, randomised controlled trials (which were not part of a review), narrative reviews and rapid reviews were excluded. Both systematic reviews of randomised controlled trials and observational studies were eligible for inclusion. To be considered for inclusion the review had to compare staples with sutures as a closure procedure. Reviews in which the stapler was not being used as a closure device were excluded. Reviews comparing different suture materials, different staples, wound line reinforcement or adhesive strips were not included, unless they also reported comparisons of staples with sutures. We also excluded reviews for which it was not possible to isolate the effect of staples and sutures, due to the involvement of differing additional procedures in each arm.

Populations of interest were those requiring surgical closure during any operative therapy either for wound or internal closure.

### Preliminary Data Abstraction

For each review meeting the inclusion criteria data were abstracted independently by two reviewers [KH and MP]. All data was compared and identified anomalies rectified by mutual agreement. Data were obtained exclusively from the systematic reviews and we did not obtain the primary study reports. Data abstracted from each systematic review included surgery type, whether the comparison was for an internal or wound closure, year published, number of studies, whether the studies were RCTs or observational studies, and the number of observations randomised by arm.

### Assessment of Data Quality

For each review we then assessed quality and risk of bias using the AMSTAR score, a tool to assess the methodological quality of systematic reviews, with independent assessment by two reviewers (KH and MP) [23]. Risk of bias for the primary studies was not assessed. However, we did record whether and how the reviews had assessed the quality of the primary studies and which method had been used (for example the Cochrane risk of bias tool).

### Exclusion of Duplicate Primary Studies

Reviews were then screened to exclude systematic reviews with duplicate primary studies. If duplicate primary studies were identified, then we selected the review to be included according to the following (ordered) preference criteria: the availability of numerical data or results (that is, reviews which did not provide summary results or numerical data which could be used to produce treatment effects were not given preference); the highest AMSTAR score (Quality assessment tool for systematic reviews); including RCTs only, or providing results so that summary treatment effects from RCTs only could be used; most recent date of publication; larger number of studies and observations included. These criteria were important where more than one systematic review had been published within a specialty. Assessments were made independently for each outcome, so that if two reviews, with duplicate primary studies, reported on different outcomes (below), then both reviews were eligible for inclusion.

### Outcomes

Principal outcomes were surgical site infection, post surgical complications, operating time and length of stay. The intention was to classify infections as superficial or deep, but insufficient data were available for this distinction to be used. Post surgical complication was dehiscence for skin to skin closures and leak for internal anastomosis, unless the authors of the review had pre-specified the post surgical complication to be something different. Preference was given to outcomes recorded within 30 days of the procedure, but other follow-up times were included where necessary.

### Data Abstraction for Non Duplicate Reviews

For each systematic review assessed as containing non duplicate primary studies, and for the four outcomes considered, summary treatment effect estimates were then abstracted, along with standard error, confidence intervals, and the number of studies, observations and number of events contributing to the analysis.

The principal measure of effect for binary outcomes was the odds ratio (but we also used relative risk if this was the only measure of effect reported); and the mean difference for continuous outcomes. Analyses reported as being by intention to treat were given preference, but other results were used if this was all that was reported.

Where the original reviews did not report a meta-analysis of results, we performed this ourselves where the data were available (to obtain an estimate of the pooled odds ratio, or mean difference for continuous outcomes, we used the inverse variance method with random effects for I^2^>40% and fixed effects otherwise). Further details are in appendix S2.

### Synthesis of Results

Initial data exploration consisted of summarising all the reported summary treatment effect estimates, number of included studies and total number of observations for each review. These data are presented in a Forest plot where each stem represents a systematic review for a particular surgery type. Results for post surgical complications and surgical site infection are stratified by whether the review was for an internal or skin to skin closure and by whether the review included both RCTs and observational studies, or just RCTs. For length of stay and operating time, there were an insufficient number of reviews to allow stratification.

### Quantitative Data Synthesis

The primary aim of this review of reviews is to provide a synthesis across surgery types, rather than to provide pooled treatment effects. However, for a subset of reviews deemed to have moderate clinical and statistical heterogeneity we provide pooled (across surgery types) treatment effects. Before pooling both statistical and clinical heterogeneity were explored.

Statistical heterogeneity in treatment effect estimates between reviews was explored using the I-squared statistic; clinical heterogeneity explored by type of closure procedure (internal or skin to skin); and design heterogeneity explored using AMSTAR scores and by stratifying by reviews which included only randomised controlled trials. We have produced pooled analyses across reviews including only RCTs, and, once again, stratified by closure type (internal or skin). Where reviews exhibited considerable statistical heterogeneity (I^2^>75%) results were not pooled.

A formal quantitative data synthesis was undertaken using a two step frequentist approach to a panoramic meta-analysis [22]. This method provided a single pooled estimate of the odds ratio for binary outcomes (staples v sutures), and mean differences for continuous outcomes, over all reviews, along with estimates of degree of heterogeneity between reviews. This allows for both between study variability (if random effects meta-analysis was used in the original review) and between review variability (using random effects), but does assume exchangeability of treatment effects. Evidence of funnel plot asymmetry was assessed using both the funnel plot and the Egger test using a conservative P-value of 0.1 to acknowledge the low power of this test.

### Ethical Approval

This is a systematic review and no ethical approval was therefore needed.

## Results

### Search

**Figure 1:**
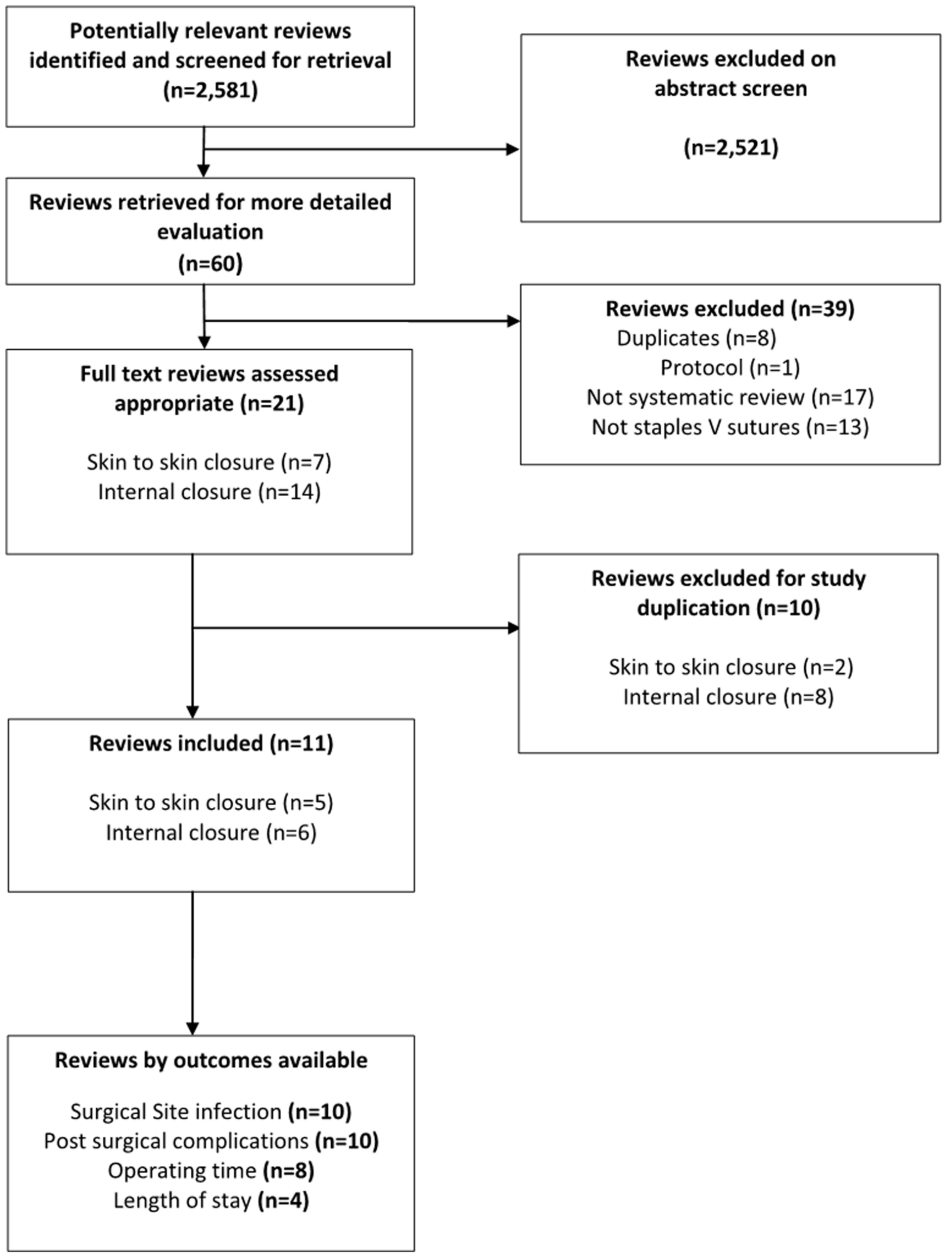
Flow diagram of reviews identified by search and those included in the analysis.

The search identified 2,581 potential reviews (Figure 1). Of these, 2,521 were excluded on the basis of an abstract screen for reasons including duplicates, studies clearly not comparing staples vs. sutures; or studies that were not systematic reviews (being primary studies, consensus statements, or rapid reviews); related reviews but not comparisons of staples and sutures (including haemorrhoidectomy and pancreatic remnant where the comparison group was not sutured, skin adhesives, staple line re-enforcement, mesh with no fixation). The remaining 60 articles were obtained in full. Of these, a further 39 were excluded, for similar reasons to those quoted above.

The 21 systematic reviews [24]–[44] meeting the inclusion criteria, before exclusions for duplication of primary studies are presented in Table 2. These reviews included both skin to skin (n = 7) and internal closures (n = 14). Skin to skin closures included the specialties of obstetrics and gynaecology (n = 4); orthopaedics (n = 1); cardio-thoracics (n = 1); and one review which included synthesis over multiple surgery types, including obstetrics and gynaecology, general surgeries, emergency procedures, head and neck surgeries and vascular surgeries (n = 1). Internal closures included the specialties of colorectal surgery (n = 7); oesophageal surgery (n = 5) and appendiceal stump (n = 2).

**Table 2:** Review and primary study quality.

Review Details and Assessment of Review Quality						Assessment of primary study quality	
Closure Type	Specialty	Surgery Type	Author and Reference	Year	AMSTAR Score	Method	Median (IQR) (unless stated otherwise)
Skin to skin	Obstetrics and gynaecology	Abdominal hysterectomy	Boesch [26]	2009	3	None	
		**Caesarean section**	**Tuuli ** [**24**]	**2011**	**9**	**PEDro**	**8 ** [**8]**, [9]
		Caesarean section	Alderdice [23]	2003	8	Cochrane handbook 2000	All adequate concealment
		Caesarean section	Clay [25]	2011	7	Jadad	range 2–3
	Other	**Multiple surgery types** *	**Iavazzo ** [**46**]	**2011**	**4**	**Jadad**	**3 ** [**2]**, [3]
		**Orthopaedic**	**Smith ** [**41**]	**2010**	**9**	**PEDro**	**3.5 ** [**2]**, [5]
		**Vein Graft following CABG**	**Biancari ** [**35**]	**2010**	**9**	**Cochrane risk of bias**	**moderate**
**Internal**	Lower gastrointestinal surgery	Colorectal anastomosis	Lustosa [28]	2002	2	None	
		**Colorectal anastomosis**	**Matos ** [**29**]	**2001**	**6**	**Cochrane handbook 1999**	**unclear**
		Colorectal anastomosis	MacRae [27]	1998	4	6-point system(6 best)	mean 5.2 range(3–6)
		**Ileocolic anastomoses**	**Choy ** [**30**]	**2011**	**9**	**Cochrane risk of bias**	**low**
		**Ileal pouch anal anastomosis**	**Lovegrove ** [**31**]	**2006**	**7**	**Newcastle-Ottawa Scale**	**6 [4.5,8]**
		Ileal pouch anal anastomosis	Schluender [32]	2006	4	None	
		**Loop ileostomy closure**	**Leung ** [**33**]	**2008**	**7**	**Jadad (and Levels of Evidence)**	**1.5 ** [**1]**–[2]
	Upper gastrointestinal surgery	**Gastro-Oesophageal anastomosis**	**Markar ** [**39**]	**2011**	**6**	**Jadad**	**2 ** [**2]**, [3]
		Gastro-Oesophageal anastomosis	Urschel [37]	2001	3	Jadad	range 2–3
		Gastro-Oesophageal anastomosis	Korolija [40]	2008	1	None	
		Gastro-Oesophageal anastomosis	Kim [38]	2010	1	None	
		Gastro-Oesophageal anastomosis	Beitler [36]	1998	0	None	
	General surgery	**Appendiceal stump**	**Kazemier ** [**44**]	**2006**	**4**	**Jadad**	**2 [0.5,3.5]**
		Appendiceal stump	Sajid [45]	2009	6	Other	moderate

**Bold**: selection for inclusion in synthesis and pooled analysis; PEDro: Physiotherapy Evidence Database critical appraisal tool out of 11 (best); Jadad score out of 5 (best); Newcastle-Ottawa scale out of 9 (best); Levels of Evidence score range 1a (best)-6 (worst); AMSTAR score out of 11 (best); Other: authors own criteria; CABG: Coronary Artery Bypass Graft;

* includes synthesis within five surgery types (obstetrics and gynaecology, general, emergency, head and neck and vascular) for infection; blank indicates it was not possible to extract this data from the review.

### Exclusions for Duplicate Primary Studies

Of these 21 systematic reviews ten were excluded from the analysis due to duplication of primary studies within reviews. Details are provided in the appendix S1 and in Table S1.

### Included Reviews

Details of the thirteen remaining reviews (from 11 distinct publications) including 13,661 observations are presented in Table 3. These reviews, published between 2001 and 2011, included a median of 5 (IQR 3–6) randomised controlled trials and between 0 and 15 (median 0 IQR 0–1) observational studies. The median number of observations included within the reviews was 762 (IQR: 322–1233), split between sutures (median IQR 465 (168–684)) and staples (median 384 IQR (147–487)). Ten of the reviews reported data on surgical site infection; 10 on complications; 4 on length of stay; and 8 on operating time. The complications for the skin to skin closure reviews were dehiscence (3) and hematoma (1); and for the internal closure reviews were anastomotic leak or complication (5), and post-operative ileus (1).

**Table 3:** Summary of included systematic reviews.

	Publication Details			Number of Trials and Number of Observations					Outcomes Reported			
Closure Type	Surgery Type	Author and Reference	Year	No. of RCTs	No. of Other Studies	Total Obs.	Total Sutures	Total Staples	Surgical Site Infection	Post surgical complications	Operating Time	Length of Stay
**Skin to skin**	Abdominal hysterectomy	Boesch [26]	2009	1^$4^	0	60	30	30		Hematoma		
	Caesarean section	Tuuli [24]	2011	5	1^$6^	1487	684	803	Yes	Dehiscence	Yes*	
	Orthopaedic	Smith [41]	2010	3	3^$3^	683	333	351	Yes^RR^	Dehiscence^RR^		
	Vein graft following CABG	Biancari [35]	2010	3^$5^	0	322	174	148	Yes^RR^	Dehiscence^RR^		
	Emergency procedures	Iavasso [46]	2011	2	0	269	93	96	Yes			
	General surgeries^$2^	Iavasso [46]	2011	5	0	936	550	386	Yes		Yes	
	Multiple surgery types	Iavasso [46]	2011	5	0	289	145	144			Yes^$2^	
**Internal**	Ileocolic anastomosis	Choy [30]	2011	7	0	1125	684	441	Yes	Overall leak	Yes	Yes
	Ileal pouch anal anastomosis	Lovegrove [31]	2006	6	15	4183	2699	1484	Yes	Anastomotic leak		
	Colorectal anastomosis	Matos [29]	2001	9	0	1233	611	622	Yes	Anastomotic leak	Yes	Yes
	Loop ileostomy closure	Leung [33]	2008	2	4	1965	1561	404	Yes^RR^	Anastomotic complications^RR^	Yes	Yes*
	Gastro-Oesophageal anastomosis	Markar [39]	2011	9	0	762	381	381		Anastomotic leak	Yes	
	Appendiceal stump	Kazemier [44]	2006	4	0^$1^	519	279	238	Yes	Post-operative ileus	Yes	Yes

* Outcome of operating time for caesarean sections was taken from review 25 (see text for details): $1: one observational study was mentioned in the paper but not described clearly; CABG: Coronary Artery Bypass Graft; RR reported relative risk (default is odds ratio); $2: includes 66 duplicate observations with review 25; $3 observational studies are not included for the outcome surgical site infection; $4∶15 studies meet the review inclusion criteria but only 1 was a staples v. sutures comparison; $5∶4 RCTs identified but one excluded later by the authors as not being a staples V. sutures comparison; $6∶1 observational study not included in results; Total obs.: is the total number of randomised observations over all trials which reported staples v. sutures (it excludes any non staples v. Sutures comparison, but includes both observational and randomised studies).

The AMSTAR scores (Table 2) for systematic reviews included in the analysis (median of 7 (IQR: 4 to 9)) were generally higher than those of the reviews were excluded due to duplication of primary studies (median of 3.5 (IQR: 1 to 6)). There was wide variability in the quality of the primary studies within the reviews and, due to reporting of many different scales, it was not possible to quantify these differences.

### Variation across Specialties

**Figure 2:**
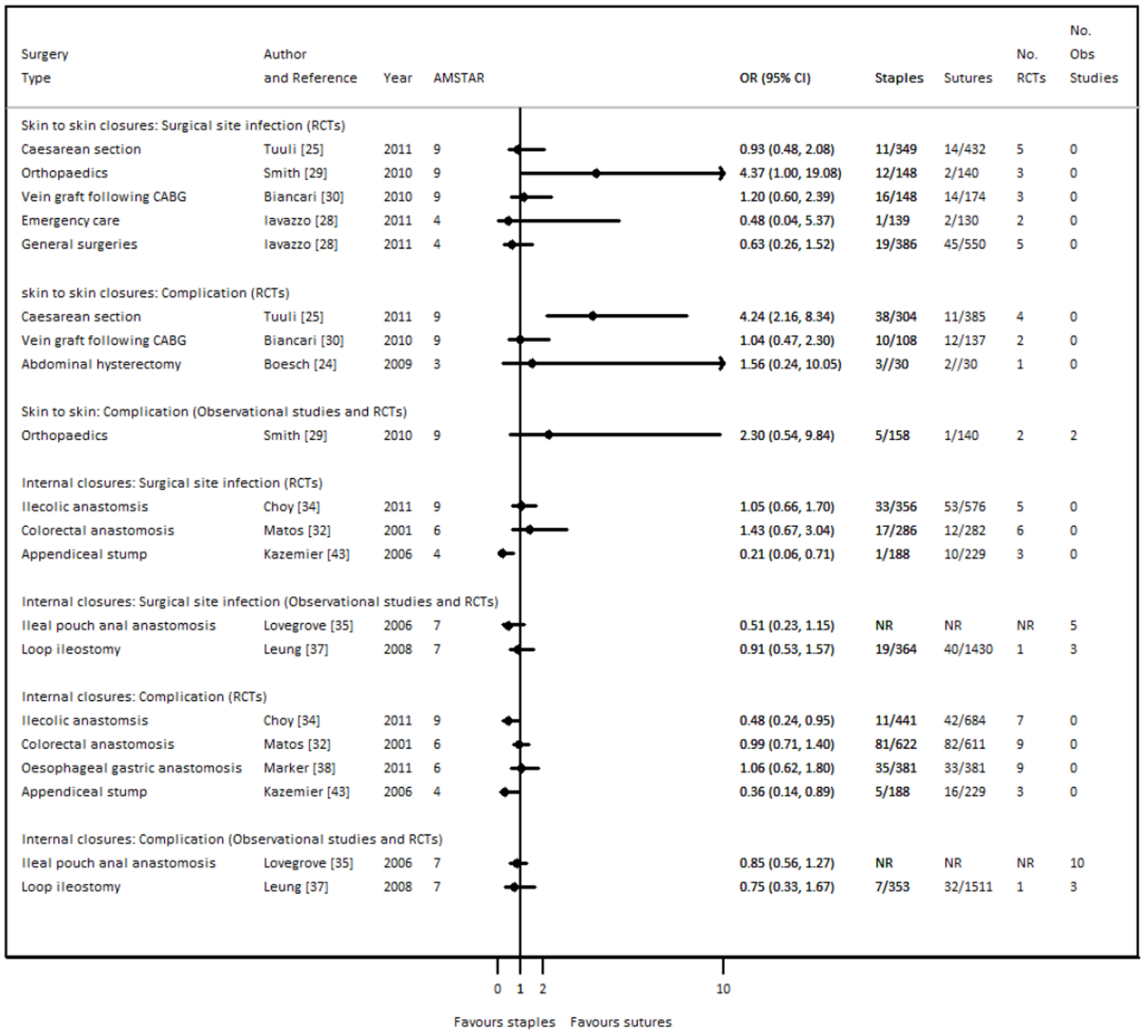
Summary of surgical site infection and post-operative complications (RRs or ORs) by surgery types and study types.

Estimates of treatment effects from each included review are presented in Figure 2 for surgical site infection and post surgical complication. There is wide variation in effect sizes between specialties, with some finding a statistically significant and beneficial effect of staples and some finding a statistically significant benefit of sutures.

For example, in orthopaedic surgery staples are associated with a statistically significant increase in surgical site infection (OR 4.37 95% CI 1.00, 19.08); and also with increased post surgical complications in caesarean section (OR 4.24 95% CI 2.16, 8.34). Yet, for appendicial stump, staples are associated with a statistically significant reduction in surgical site infection (OR 0.21 95% CI 0.06, 0.71); and the post operative complication of ileus (OR 0.36 95% CI 0.14, 0.89); and also the post operative complication of overall leak in ilecolic anastomosis (OR: 0.48 95% CI 0.24, 0.95).

There are no obvious differences between reviews which included only RCTs and those which included both RCTs and observational studies. Estimates from reviews for internal closures showed a tendency towards providing more precise estimates (i.e. narrower confidence intervals). For those reviews which included RCTs only, low heterogeneity was observed between reviews for the outcome surgical site infection in skin closure procedures (I^2^ 28%) and moderate to high heterogeneity (I^2^ 72%) was observed between reviews for the outcome post surgical complications. For internal procedures, and again for the subset of reviews which included RCTs only, there was a moderate to high level of heterogeneity (I^2^ 72% for surgical site infection and I^2^ 59% for post surgical complication).

**Figure 3:**
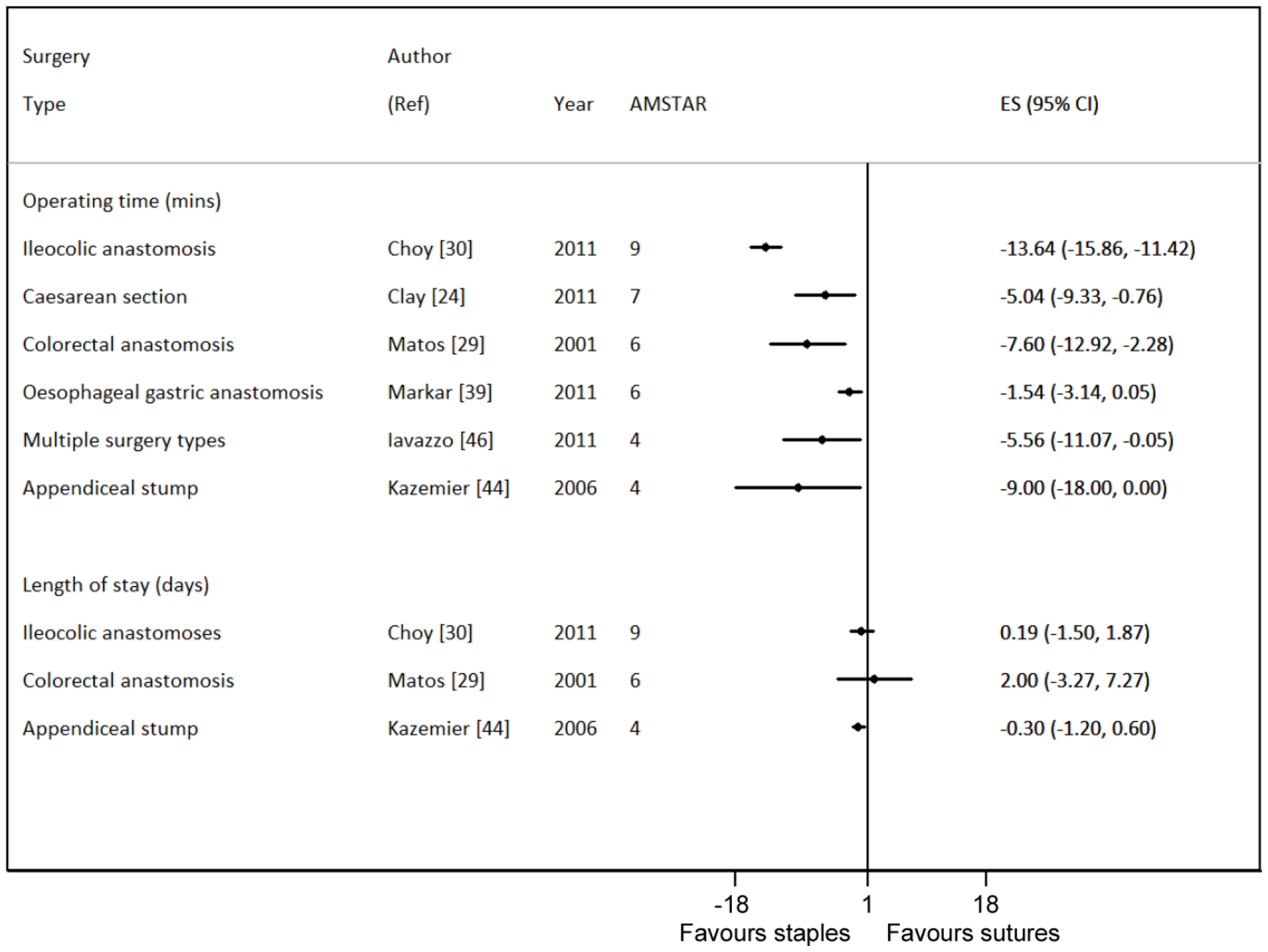
Summary of operating time and length of stay (mean differences) by surgery types and study types.

For the outcomes of length of stay and operating time pooled estimates within specialties are presented in Figure 3. There is again wide variation between specialties, but for the outcome length of operating time, all show a preference towards staples. Whilst there was no evidence of statistical heterogeneity between the reviews for difference in length of stay (I^2^ 0%), there was considerable heterogeneity between the reviews for differences in operating time (I^2^ 94%).

### Pooled Estimates across Specialties

For the subset of reviews which included RCTs only, we pooled across surgery types, with stratification for internal and skin to skin closures (Table 4).

**Table 4:** Pooled estimates across reviews for surgical site infection and post surgical complication for RCTs only.

Surgery Type		Author and Reference	Complication	Year	AMSTAR Score	No. RCTs	Staples (r/n)	Sutures(r/n)	OR	LCI	UCI	% weight
	**Skin to skin closure: surgical site infection**											
Caesarean section*		Tuuli [24]		2011	9	5	11/349	14/432	0.93	0.48	2.08	30
Orthopaedics*		Smith [41]		2010	9	3	12/148	2/140	4.37	1.00	19.08	11
Vein graft following CABG		Biancari [35]		2010	9	3	16/148	14/174	1.20	0.60	2.39	32
Emergency care		Iavazzo [46]		2011	4	2	1/139	2/130	0.48^$1^	0.04	5.37	4
General surgeries		Iavazzo [46]		2011	4	5	19/386	45/550	0.63^$1^	0.26	1.52	23
									**1.05**	**0.63**	**1.77**	**I^2^ = 28% Egger P-value 0.77**
	**Skin to skin closure: post surgical complications**											
Caesarean section*		Tuuli [24]	Dehiscence	2011	9	4	38/304	11/385	4.24	2.16	8.34	41
Vein graft following CABG		Biancari [35]	Dehiscence	2010	9	2	10/108	12/137	1.04	0.47	2.30	39
Abdominal hysterectomy		Boesch [26]	Dehiscence	2009	3	1	3/30	2/30	1.56^$2^	0.24	10.05	20
									**2.02**	**0.69**	**5.86**	**I^2^ = 72% Egger P-value 0.91**
	**Internal closure: surgical site infection**											
ileocolic anastomosis		Choy [30]		2011	9	5	33/356	53/576	1.05	0.66	1.70	42
Colorectal anastomosis		Matos [29]		2001	6	6	17/286	12/282	1.43	0.67	3.04	35
Appendiceal stump		Kazemier [44]		2006	4	3	1/188	10/229	0.21	0.06	0.71	23
									**0.80**	**0.35**	**1.85**	**I^2^ = 72% Egger P-value 0.52**
		**Internal closure: post surgical complications**										
ileocolic anastomosis		Choy [30]	Anastomotic leak	2011	9	7	11/441	42/684	0.48	0.24	0.95	22
Colorectal anastomosis		Matos [29]	Anastomotic leak	2001	6	9	81/622	82/611	0.99	0.71	1.40	35
Gastro-Oesophageal anastomosis		Marker [39]	Anastomotic leak	2011	6	9	35/381	33/381	1.06	0.62	1.80	27
Appendiceal stump		Kazemier [44]	Post op ileus	2006	4	3	5/188	16/229	0.36	0.14	0.89	16
									**0.74**	**0.47**	**1.16**	**I^2^ = 59% Egger P-value 0.15**

CABG: Coronary Artery Bypass Graft; LCI: Lower Confidence Interval; UCI: Upper Confidence Interval;

* Original review included both RCTs and observational studies but results reported here pertain only to RCTs. AMSTAR score is an assessment of review quality (0–11 best), see text for details; Egger is test for publication bias (see text); % weight is weight from a random effects panoramic meta-analysis; $1: results in paper were staples v sutures, results here are sutures v staples; S2: no pooling in paper we computed this estimate.

There were five reviews which reported on skin to skin closures and which included RCTs only; and three reviews which reported on post surgical complications. After pooling over reviews, there was no evidence of a difference in either surgical site infection (OR 1.05 95% CI 0.63, 1.77); and whilst there was some indication that staples resulted in increased odds of post surgical complications, the 95% confidence interval was wide indicating considerable uncertainty (OR 2.02 95% CI 0.69, 5.86).

For internal procedures, three reviews including only RCTs reported on surgical site infection and four reviews on post surgical complications. There was again no evidence of a difference between staples and sutures for the outcome surgical site infection (OR 0.80 95% CI 0.35, 1.85); nor for the outcome post surgical complications (OR 0.74 95% CI 0.47, 1.16), although for this outcome there was evidence of publication bias (Egger test P-value 0.15).

Six reviews, including only RCTs, reported on operating time and three on length of stay (Table 5). Pooling over reviews, for length of stay, no clear differences were seen between staples and sutures, with staples resulting in an average mean length of stay reduction of 0.1 day (95% CI −0.9, 0.6). However, again, there was some indication of publication bias among reviews (Egger test P-value 0.05).The degree of statistical heterogeneity was too large to consider pooling over reviews for operating time, but every review which reported on this found that staples resulted in a reduction in mean operating time (between 1.5 minutes (95% CI −3, 0) for oesophageal gastric anastomosis and 14 minutes (95% CI −16, −11) for ilecolic anastomsis).

**Table 5:** Pooled estimates across reviews for length of stay and operating time.

Surgery	Author and Reference	Year	No. Obs.	No. in analysis	AMSTAR	Mean Difference	LCI	UCI	% weight
**Operating time (minutes)**									
Ileocolic anastomosis	Choy [34]	2011	1125	255 (23)	9	−13.64	−15.86	−11.42	
Caesarean section	Clay [27]	2011	877	811 (92)	7	−5.05	−9.33	−0.76	
Colorectal anastomosis	Matos [32]	2001	1233	159 (13)	6	−7.60	−12.92	−2.28	
Gastro-Oesophageal anastomosis	Markar [38]	2011	762	569 (75)	6	−1.56	−3.14	0.05	
Multiple surgery types	Iavazzo [28]	2011	281	281(100)	4	−5.56	−11.07	−0.05	
Appendiceal stump	Kazemier [43]	2006	467	517 (90)	4	−9.00	−18.00	0.00	
			**3635**	**2592(71)**					**I^2^ = 94% Egger P-value = 0.64**
**Length of stay (days)**									
Ileocolic anastomosis	Choy [34]	2011	1125	424 (37)	9	0.19	−1.50	1.87	21.7
Colorectal anastomosis	Matos [32]	2001	1233	159 (13)	6	2.00	−3.27	7.27	2.2
Appendiceal stump	Kazemier [43]	2006	427	367 (86)	4	−0.30	−1.20	0.60	76.1
			**2785**	**950 (34)**		**−0.13**	**−0.93**	**0.64**	**I^2^ = 0% Egger P-value = 0.05**

LCI: Lower Confidence Interval; UCI: Upper Confidence Interval. AMSTAR score is an assessment of review quality (0–11 best), see text for details; Blank indicates it was not possible to extract this data from the review; % weight is weight from a random effects panoramic meta-analysis; No. Obs. refers to the intention to treat population and includes all patients randomised over all of the included studies (excludes observational studies if the analysis only includes the subset of randomised studies).

## Discussion

### Findings

We have reported the first systematic review of systematic reviews of studies comparing staples and sutures following any operative skin to skin or internal wound closure. Twenty-one relevant systematic reviews were identified, from which we carefully excluded duplicate studies. For the 11 reviews identified after excluding reviews containing duplicate primary studies, there was a clear indication, that although operating times varied considerably across specialties, on average, staples result in decreased length of operating time: between 1.5 minutes (95% CI −3, 0) for gastro-oesophageal anastomosis and 14 minutes (95% CI −16, −11) for ileocolic anastomosis. For the clinical outcomes of surgical site infection and post surgical complication there was no consistent evidence that one method out performs the other across all surgery sites.

In orthopaedic surgery staples were found to be associated with a statistically significant increase in surgical site infection (OR 4.37 95% CI 1.00, 19.08); and also with increased post surgical complications in caesarean section (OR 4.24 95% CI 2.16, 8.34). Given that this review was of high quality (AMSTAR score 9) then arguably the evidence suggests that within orthopaedic surgery sutures would seem to lead to better patient outcomes. For appendicial stump, staples were found to be associated with a statistically significant reduction in surgical site infection (OR 0.21 95% CI 0.06, 0.71); and the post operative complication of ileus (OR 0.36 95% CI 0.14, 0.89). However, this review is of lower quality (AMSTART score 4) and to the reliability of this finding more uncertain. Finally for the post operative complication of overall leak in ilecolic anastomosis staples were again found to be protective (OR: 0.48 95% CI 0.24, 0.95), although this was not also true for the outcome of surgical site infection (OR: 1.05 95% CI: 0.66, 1.77).

For all other surgery types the evidence was inconclusive with wider confidence intervals including the possibly of preferential outcomes for surgical site infection or post surgical complication for either staples or sutures.

The primary aim of this review was to provide a synthesis across surgery types, as opposed to a meta-analysis. For both skin to skin closures and internal procedures, whilst there was some evidence of statistical heterogeneity between the reviews for both of the outcomes considered (surgical site infection and post surgical complications), these levels would not normally be considered prohibitive of pooling under a conventional meta-analysis. We therefore additionally computed pooled (across surgery types) treatment effects for the subset of reviews which included RCTs only.

For skin to skin closures, for both surgical site infection and post surgical complication, there is no evidence to suggest whether staples or sutures result in improved outcomes. Five reviews with minimal heterogeneity between reviews (I^2^ 28%) with 2,596 observations gave an OR 1.05 (95% CI 0.63 1.77) for surgical site infection; three reviews (I^2^ 72%) with 1,164 observations gave an OR 2.02 (95% CI 0.69–5.86) for post surgical complications.

For internal closures, again the statistical levels of heterogeneity did not suggest that reviews should not be pooled. However, whilst pooling results for post surgical complications might be legitimate due to only moderate heterogeneity (I^2^ 59%), evidence of small study, or publication type bias suggested that this pooled estimate could be prone to bias (OR: 0.74 95% CI 0.47–1.16, P-value for Egger test 0.15).

Few reviews reported on length of stay, but those three reviews that did (I^2^ 0%, including 950 observations) showed a significant reduction in length of stay of mean ½ day (95% CI: 0.1 to 1 day) with staples but, again, showed evidence of publication bias (P-value for Egger test 0.05).

### Pooling Systematic Reviews of Systematic Reviews

In 1955 Stein showed, perhaps paradoxically, that it can be prudent to take account of external evidence in quantifying treatment effects [45]. Sometimes referred to as shrinkage, this is not only a Bayesian phenomenon, and comes about because of the increase in precision that is obtained when incorporating external information, and shrinkage towards the grand mean. So, for example, in evaluating local prevalence of disease, using information from not too distant localities will result in a more precise and possibly less biased estimate. Clearly the improvements in precision stem from the increased sample size. Perhaps less obviously, the reduction in bias results from a dilution of large (perhaps large chance findings) and small (perhaps small chance findings) effects towards the underlying average.

However, shrinkage is not always appealing. A large survey in South Africa on prevalence of HIV might well add to the precision of that from a small UK based survey, but shrinkage would clearly be undesirable - prevalence of HIV in South Africa tells us little about prevalence in the UK. In statistical terms these two prevalence’s are not exchangeable. That is, shrinkage is only desirable when the quantities estimated in various studies are considered exchangeable. So, in a conventional meta-analysis it is sometimes considered that the degree of heterogeneity between studies may preclude pooling of study estimates. In statistical terms this means that the treatment effects are not exchangeable.

### Limitations

The issue of whether the treatment effects of staples v. sutures from two different surgery types are sufficiently exchangeable to warrant pooling across surgery types, is both an issue of statistical heterogeneity and clinical heterogeneity. Clean and contaminated surgery types are clearly clinically heterogeneous, and rates of surgical site infection differ between the two [46]. This however in itself does not automatically preclude the pooling of treatment effects.

Treatment effects will be statistically heterogeneous if the effect of the treatment in different surgery types differ substantially. The effectiveness of many treatments indeed vary by severity and so the effect of sutures and staples may well vary between surgery types. Other sources of heterogeneity are surgeon ability, which may vary by surgical specialty; and underlying morbidity. Whilst these are real causes for concern, it might be argued that the identification of sub-group effects (i.e. differential effects of staples and sutures across surgical specialties) should not proceed the identification of the overall effect.

### Evidence for Small Study Bias

Some indication of small study (or publication) bias was evident in this review. Funnel plot asymmetry might be a consequence of small study bias (often referred to as publication bias), methodological quality, or might be due to the heterogeneity of reviews. Small study bias would suggest selective reporting of smaller reviews; whilst heterogeneity induced funnel plot asymmetry would suggest variation in efficacy by study size, which in turn might be due to variation in effect by speciality. All of these are tenable: selective reporting of complications would occur if reviewers selected complications to report based on their statistical significance; methodological quality undoubtedly varied, possibly by review size; and the moderate heterogeneity observed between reviews suggest some varying of effect by specialty. Different specialties reported different outcomes, many of which could be construed as post surgical complications. The most noticeable indication of possible small study bias was for the complication outcome. Some systematic reviews reported post surgical complication directly whilst others reported various complications. For these reviews, some subjectivity was involved in selecting one of the reported adverse outcomes as the complication. Bias due to selective reporting of outcomes, is an established source of bias, and is also possible here.

### Conclusion

Staples are frequently used to restore epithelial integrity both in skin closure and in intestinal reconstruction. Many randomised controlled trials, and systematic reviews, have tried to resolve the important question of whether staples or sutures improve outcomes. The evidence strongly suggests that use of staples results in reduced operative time. Reduction in operative time has the potential to reduce tissue handling and associated tissue injury, suggesting staples might well lead to improved patient outcomes, but we did not find any evidence of this. For internal procedures, additional potential for mitigating adverse events is likely to come from the ability of the closure procedure (i.e. staples or sutures) to reduce intraoperative contamination. But, again we were unable to find any support for preferential use of either method to improve patient outcomes of surgical site infection or post surgical complications for patients undergoing internal anastomosis.

## Supporting Information

Table S1 **Summary of excluded systematic reviews.**
(DOCX)

Appendix S1 **Exclusions for duplication of primary studies.**
(DOCX)

Appendix S2 **Computation of within review pooled estimates.**
(DOCX)

Checklist S1 **PRISMA checklist.**
(DOC)
