# Supplementary material for: A Systematic Review of Systematic Reviews and Panoramic Meta-Analysis: Staples versus Sutures for Surgical Procedures
Source: PLoS One. 2013 Oct 7;8(10):e75132. doi: 10.1371/journal.pone.0075132 (PMC3792070; doi:10.1371/journal.pone.0075132)
Supplement: Table S1 — Summary of excluded systematic reviews. (DOCX) [file pone.0075132.s001.docx]

|  | **Publication Details** | | | **Number of Trials and Number of Observations** | | | | | **Outcomes Reported** | | | |
| --- | --- | --- | --- | --- | --- | --- | --- | --- | --- | --- | --- | --- |
| **Closure Type** | **Surgery Type** | **Author and Reference** | **Year** | **No. of RCTs** | **No. of Other Studies** | **Total**  **Obs.** | **Total**  **Sutures** | **Total**  **Staples** | **Surgical Site Infection** | **Post surgical complications** | **Operating Time** | **Length of Stay** |
| **Skin to skin** | Caesarean section | Clay [27] | 2011 | 5 | 0 | 877 | 492 | 385 |  | Composite wound complication^$4^ | Yes^$2^ |  |
|  | Caesarean section | Alderdice [26] | 2003 | 1 | 0 | 66 | 32 | 34 | Yes | Dehiscence | Yes |  |
| **Internal** | Colorectal anastomosis | Lustosa [31] | 2002 | 9 | 0 | 1233 | 611 | 622 | Yes | Anastomotic leak | Yes | Yes |
|  | Colorectal anastomosis | MacRae [33] | 1998 | 13 | 0 | 2256 | 1249 | 1007 | Yes | Anastomotic leak |  |  |
|  | Ileal pouch anal anastomosis | Schluender [36] | 2006 | 4 | 0 | 184 | 86 | 98 |  |  |  |  |
|  | Gastro-Oesophageal anastomosis | Urschel [39] | 2001 | 5 | 0 | 467 | 231 | 236 |  | Anastomotic leak |  |  |
|  | Gastro-Oesophageal anastomosis | Korolija [40] | 2008 | 3^$1^ | 2 | 1123 | 532 | 591 |  | Anastomotic leak* | Yes* |  |
|  | Gastro-Oesophageal anastomosis | Kim [41] | 2010 | 8 | 0 | 710 | 356 | 354 |  | Anastomotic leak* | Yes* |  |
|  | Gastro-Oesophageal anastomosis | Beitler [42] | 1998 | 4 | 3 | 2380 | 1264 | 1116 |  | Anastomotic leak* |  |  |
|  | Appendiceal stump | Sajid [44] | 2009 | 5 | 0 | 622 | 359 | 263 |  | Perioperative complication*^$5^ | Yes* | Yes* |

**Table A1: Summary of excluded systematic reviews**

* no meta-analysis or meta-analysis which is presented in graphical format only; $1: Five systematic reviews were also identified in this review and one case series which are not included here; $2: this review is included for the outcome operating time only (see text for an explanation); $4: includes dehiscence, wound infection, seroma and hematoma; $5: includes wound infection, abscess, and other; blank indicates it was not possible to extract this data from the review. Total Obs. Here includes all those patients randomised in both observational and RCTs.
