# Supplementary material for: A Systematic Review of Systematic Reviews and Panoramic Meta-Analysis: Staples versus Sutures for Surgical Procedures
Source: PLoS One. 2013 Oct 7;8(10):e75132. doi: 10.1371/journal.pone.0075132 (PMC3792070; doi:10.1371/journal.pone.0075132)
Supplement: Appendix S1 — Exclusions for duplication of primary studies. (DOCX) [file pone.0075132.s002.docx]

**Appendix S1: Exclusions for duplication of primary studies**

Four reviews were identified for caesarean section [25-28] and one for abdominal hysterectomy [24]. The four reviews for caesarean section showed considerable overlap. We selected for inclusion the review with the higher AMSTAR score (9) [25]; however since this review did not report a pooled result for the outcome operating time, we additionally included the review with the lower AMSTAR score [27] which did report the outcome operating time but only for this outcome. The abdominal hysterectomy review [24] was included as it did not show any duplication with the primary studies included in the other obstetric reviews.

Four reviews were published on colorectal anastomosis, including ileocolic anastomosis [31-34]. Two of these were by the same authors and contained identical primary studies [31, 32]. Of these we selected for inclusion the review that scored the higher AMSTAR score [32], this happened to be the review that was published earlier. This left three remaining reviews [32-34] which showed considerable overlap of included primary studies, with one of the reviews [33] including almost all of those in the other two reviews [32, 34] (which did not overlap). Again, we selected for inclusion the two reviews of higher quality [32, 34] as opposed to the larger, but lower quality review [33]. Two reviews were identified for ileal pouch-anal anastomosis, one [36] of which was excluded since all studies were included in the other higher quality review [35], despite both reviews being published in the same year.

Five reviews were identified for oesophageal gastric anastomosis [38-42]. One of these reviews [40] did not include a meta-analysis and neither provided any data from which pooled results could be obtained. Three reviews published in 1998, 2001 and 2010 [42, 39, 41] were excluded since all but one of the primary studies were included in a later, and again higher quality, review published in 2011 [38].

Two reviews were available for appendiceal stump [43, 44]. One of these reviews [44], which also had the higher AMSTAR score, did not report numerical meta-analysis results, or numerical data to allow pooling of results, and so was thus precluded from our analysis

One review which reported on length of stay and surgical site infection reported across multiple surgery types, including obstetrics, general surgeries, emergencies, head and neck and vascular surgeries [28]. As detailed above, the obstetric and gynaecology part of this review was not included as it showed considerable overlap with another higher quality review [25]. Furthermore, two of the surgery types (head and neck and vascular) included no events in either arm and so could not contribute to any analysis. This therefore left just two surgery types (general and emergency procedures) which were selected for inclusion. For the outcome length of operating time, this review did not stratify by surgery type and whilst it did include data from one study included in the obstetric review [25], this minor overlap was not considered to be so substantial as to warrant exclusion.

**Appendix 2: Computation of within review pooled estimates**

Several reviews whilst providing numerical data did not provide pooled estimates; or if they did included trials which did not meet our inclusion or preference criteria. In these cases using methods described above, we computed pooled (over studies within a review) summary estimates. For the review on caesarean section [25] numerical meta-analysis results were provided across both observational and RCTs. Trial data within the review allowed pooling of results across RCTs only. This was also the case for the surgical site infection outcome in the orthopaedic review [29]. Only one study within the gynaecology review [24] met our inclusion criteria (as the review was across different interventions only one of which was staples V sutures) and so no pooling was necessary. For the gynecology review [24] no treatment effect was reported in the review and so we computed this. The results of this single study were entered into our analysis and no pooling was necessary [24]. Two reviews [35, 28] reported effect size estimates as sutures vs. staples, as opposed to the more conventional staples vs. sutures, to adjust for this we re-estimated using the study level data and using the method of pooling used by the authors in the review; or inverted the pooled estimates if the study level data were not available.
