# Supplementary material for: A Systematic Review of Systematic Reviews and Panoramic Meta-Analysis: Staples versus Sutures for Surgical Procedures
Source: PLoS One. 2013 Oct 7;8(10):e75132. doi: 10.1371/journal.pone.0075132 (PMC3792070; doi:10.1371/journal.pone.0075132)
Supplement: Appendix S2 — Computation of within review pooled estimates. (DOCX) [file pone.0075132.s003.docx]

**Appendix S2: Computation of within review pooled estimates**

Several reviews whilst providing numerical data did not provide pooled estimates; or if they did included trials which did not meet our inclusion or preference criteria. In these cases using methods described above, we computed pooled (over studies within a review) summary estimates. For the review on caesarean section [25] numerical meta-analysis results were provided across both observational and RCTs. Trial data within the review allowed pooling of results across RCTs only. This was also the case for the surgical site infection outcome in the orthopaedic review [29]. Only one study within the gynaecology review [24] met our inclusion criteria (as the review was across different interventions only one of which was staples V sutures) and so no pooling was necessary. For the gynecology review [24] no treatment effect was reported in the review and so we computed this. The results of this single study were entered into our analysis and no pooling was necessary [24]. Two reviews [35, 28] reported effect size estimates as sutures vs. staples, as opposed to the more conventional staples vs. sutures, to adjust for this we re-estimated using the study level data and using the method of pooling used by the authors in the review; or inverted the pooled estimates if the study level data were not available.
